# ONC201 induces the unfolded protein response (UPR) in high‐ and low‐grade ovarian carcinoma cell lines and leads to cell death regardless of platinum sensitivity

**DOI:** 10.1002/cam4.3858

**Published:** 2021-05-01

**Authors:** Marufa Rumman, Steven Buck, Lisa Polin, Sijana Dzinic, Julie Boerner, Ira S. Winer

**Affiliations:** ^1^ Department of Oncology Karmanos Cancer Institute Wayne State University Detroit MI USA; ^2^ Division of Hematology/Oncology Department of Pediatrics Children's Hospital of Michigan Detroit MI USA; ^3^ Division of Gynecologic Oncology Department of Oncology Karmanos Cancer Institute Wayne State University Detroit MI USA

**Keywords:** apoptosis, high‐grade ovarian cancer, low‐grade ovarian cancer, ONC201, unfolded protein response

## Abstract

**Objectives:**

Treatment of both platinum resistant high grade (HG) and low‐grade (LG) ovarian cancer (OVCA) poses significant challenges as neither respond well to conventional chemotherapy leading to morbidity and mortality. Identification of novel agents that can overcome chemoresistance is therefore critical. Previously, we have demonstrated that OVCA has basal upregulated unfolded protein response (UPR) and that targeting cellular processes leading to further and persistent upregulation of UPR leads to cell death. ONC201 is an orally bioavailable Dopamine Receptor D2 inhibitor demonstrating anticancer activity and was found to induce UPR. Given its unique properties, we hypothesized that ONC201 would overcome platinum resistance in OVCA.

**Methods:**

Cisplatin sensitive and resistant HG OVCA and two primary LG OVCA cell lines were studied. Cell viability was determined using MTT assay. Cell migration was studied using wound healing assay. Apoptosis and mitochondrial membrane potential were investigated using flow cytometry. Analysis of pathway inhibition was performed by Western Blot. mRNA expression of UPR related genes were measured by qPCR. In vivo studies were completed utilizing axillary xenograft models. Co‐testing with conventional chemotherapy was performed to study synergy.

**Results:**

ONC201 significantly inhibited cell viability and migration in a dose dependent manner with IC50’s from 1‐20 µM for both cisplatin sensitive and resistant HG and LG‐OVCA cell lines. ONC201 lead to upregulation of the pro‐apoptotic arm of the UPR, specifically ATF‐4/CHOP/ATF3 and increased the intrinsic apoptosispathway. The compensatory, pro‐survival PI3K/AKT/mTOR pathway was downregulated. In vivo, weekly dosing of single agent ONC201 decreased xenograft tumor size by ~50% compared to vehicle. ONC201 also demonstrated significant synergy with paclitaxel in a highly platinum resistant OVCA cell‐line (OV433).

**Conclusions:**

Our findings demonstrate that ONC201 can effectively overcome chemoresistance in OVCA cells by blocking pro‐survival pathways and inducing the apoptotic arm of the UPR. This is a promising, orallybioavailable therapeutic agent to consider in clinical trials for patients with both HG and LG OVCA.

## Graphical abstract

ONC201 can effectively overcome chemoresistance in both high‐grade and low‐grade ovarian cancer cells by blocking pro‐survival pathways and inducing the apoptotic arm of the UPR. A weekly oral dose of ONC201 lowers the tumor burden in mice. This is a promising therapeutic agent in OVCA treatment and might consider for clinical translation.

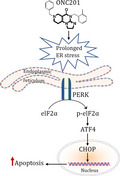

## 1 INTRODUCTION

Ovarian Cancer (OVCA) is the most lethal of all gynecologic malignancies, with an estimated 22,240 deaths in 2020 in the United States.^1^ The vast majority of all epithelial cancer is the serous subtype. According to a two‐tier system for grading epithelial serous OVCA it is classified into low grade (LG‐OVCA, 5%–10% of all epithelial OVCA) and high grade (HG‐OVCA, 90%–92% of all tumors).^2^, ^3^ Though LG‐OVCA is much less common and tumor growth is more indolent than its counterpart HG‐OVCA,^4^ both subtypes develop resistance to chemotherapy over time.^5^, ^6^ Specifically, for HG‐OVCA, patients respond upfront in ~80% to standard therapy with carboplatin and taxanes; however, ~20% are platinum refractory (i.e., progressive disease on upfront therapy) at diagnosis. Additionally, the majority of patients will recur within 2 years of primary treatment with many becoming platinum resistant at first or second recurrence.^7^, ^8^ Once platinum resistance has developed, overall response rates to chemotherapy are generally in the 10%–30% range and decrease progressively over time. Overall survival is generally in the one to 2‐year range once a patient becomes platinum resistant with no significant advances made in recent years to increase survival.^7^, ^9^ For LG‐OVCA, median survival is 99 months and 10 year ~70% given the indolent nature of disease. However, only ~50% of patients at completion of upfront surgery and platinum/taxane therapy will be free of disease and in the residual or recurrent setting, response to prototypical chemotherapy regimens is only ~4%.^3^ Therefore, identifying a novel agent to overcome chemoresistance is critical in both HG‐OVCA and LG‐OVCA.

The endoplasmic reticulum (ER) serves as a platform for cross‐communication among key cellular pathways for the maintenance of cellular homeostasis. The unfolded protein response (UPR) is the central mechanism whereby the ER in normal cells localizes the response to a stressed milieu. Via the ER trans‐membrane proteins RNA‐dependent protein kinase‐like ER eukaryotic initiation factor‐2α kinase (PERK), inositol‐requiring ER‐to‐nucleus signaling protein 1 (IRE1) and activating transcription factor 6 (ATF6), cellular stress signals are translated to downstream targets leading initially to an “alarm” phase reaction. This includes a decrease in protein translation, cell‐cycle arrest, and ultimately to an increase in specific protein translation, such as ER‐specific chaperones to assist in proper protein folding/translation and removal of improperly folded proteins via the proteasomal pathway or ER‐associated protein degradation.^10^ Tumor cells actively usurp UPR to protect the cell from unfavorable microenvironment stressors such as glucose deprivation, hypoxia. Short‐term induction of the UPR has been associated with chemotherapy resistance in vitro via upregulation of Bip/Grp78.^11^, ^12^, ^13^ One potential manner to overcome this mechanism of survival is prolonged activation, or saturation, of the UPR program via its intrinsic components or through bystander pathways in the ER. The PERK arm, primarily through ATF4‐mediated transcriptional induction of C/ebp homologous protein (CHOP/GADD153) is likely the major mediator of apoptosis and potentially autophagy‐related necrosis in prolonged activation above homeostatic/baseline levels.^14^, ^15^, ^16^ Caspases along the surface of the ER and in the cytosol, interactions with the BCl‐2 family members and transcriptional and translational upregulation of pro‐apoptotic proteins have also been implicated in addition to lysosome and Golgi machinery.^17^, ^18^ Given these findings, we hypothesize that the constitutive UPR activation, especially with preferential induction of the CHOP arm, may be an attractive mechanism to drive chemoresistant cancer cells into apoptosis.

ONC201, also known as TIC10 is a small molecule inhibitor of Dopamine Receptor D2 (DRD2) recently showing promise as a well‐tolerated anti‐cancer agent in limited Phase I and Phase II clinical studies.^19^ Dual inhibition of Akt and ERK by ONC201 in some cancer types leads to cell death via upregulation of TRAIL and induction of extrinsic apoptosis ^20^ and potentially ER stress.^21^, ^22^ Depending on cancer cell type, the exact mechanism of ONC201 can, however, differ. Here, we examine the efficacy of ONC201 in HG‐OVCA and LG‐OVCA cancer cell lines and elucidate the mechanism of action of this targeted therapeutic in ovarian cancer.

## 2 MATERIAL AND METHODS

### 2.1 Cell lines

High‐grade human ovarian cancer cells (TOV112D, OV433, SKOV3, and CaOV3) were bought from American Type Culture Collection (ATCC). TOV112D, OV433, and CaOV3 cells were cultured in Dulbecco's modified Eagle's medium (DMEM) supplemented with 10% heat‐inactivated fetal bovine serum (FBS) and 1% penicillin/streptomycin. SKOV3 cells were cultured in Roswell Park Memorial Institute 1640 (RPMI‐1640) media and TOV112D, OV433, CaOV3 cells were grown in Dulbecco's Modified Eagle Medium (DMEM). Low‐grade human ovarian carcinoma cells (VOA 4627, VOA 1312) are generous gifts from Dr Mark Carey, University of Vancouver^5^ and both of these cells were maintained in M199: MCDB 105 (1:1). All media were supplemented with 10% FBS and 1% Antibiotics. To maintain cells in vitro at 37°C in an incubator with a controlled humidified atmosphere composed of 95% air and 5% CO_2_ was used.

### 2.2 Drug and antibodies

ONC201 was utilized from Oncoceutics for in vitro and in vivo studies. ONC201 was dissolved in dimethylsulfoxide (DMSO) at 10 mM stock concentration and diluted freshly to working concentrations for all in vitro studies. For in vivo experiments, the desired dose was prepared by dissolving the drug in olive oil (diluent) for oral administration (PO) via gavage. Antibodies were purchased from Cell Signaling Technology, and Millipore Sigma; PARP (#9542), Bim (#2933), Mcl1 (#4572), CHOP (#2895), ATF4 (#11815), pAKT (#4060, Serine‐473 phosphorylation), AKT (#4691), p‐mTOR (#2971), mTOR (# 2972), pERK1/2 (#9101), ERK (#4695), Wee1 (#4936), TRAIL (#3219), and β‐actin (#A2228).

### 2.3 Cell viability assays

Cell viability evaluation was performed using the MTT (3‐(4,5‐dimethylthiazol‐2‐yl)‐2,5‐diphenyltetrazolium bromide) assay and trypan blue exclusion assay. Cells were seeded at a density of 5000–7000 cells/well in 96‐well plates following by an overnight incubation. On the following day, the media were removed and the cells were treated with either vehicle as a control or various concentrations of ONC201 following an incubation for 48, 72, and 96 h. After 48/72 h treatment, 10% of an MTT solution (5 mg/ml) was added to each well and incubated for another 2 h at 37°C to allow the formation of formazan crystals. DMSO (200 μl/well) were added to dissolve the formazan crystals that formed with constant shaking for 5 min. The absorbance was read with a microplate reader at 540 nm.^23^ In trypan blue exclusion experiment, OVCA cells were diluted in a 1:1 mixture of trypan blue (Invitrogen) and counted using the Countess II Automated Cell Counter (Invitrogen), according to the manufacturer's instructions. Three replicate wells were evaluated for each analysis and averaged.

### 2.4 Wound healing migration assay

50,000–100,000 cells, depending on the cell line, were plated on 12 well plates to obtain 95% confluent monolayer of cells after overnight incubation. Following day a linear wound was made (~0.8 inch wide) using a 200 μl pipette tip and the detached cells were washed away by Dulbecco's phosphate‐buffered saline (DPBS). Then, the monolayer was treated with the desired doses of ONC201 (0–100 μM). The cells were allowed to migrate for 8–20 h depending on the cell line. The wells were washed with DPBS and fixed in absolute methanol following crystal violet staining. The healed area with migrated cells was observed under a phase‐contrast microscope and photographed at 200X magnification.

### 2.5 Quantification of gene expression by real‐time RT‐PCR

Cells‐to‐CT kit (Thermo Fisher Scientific) was used to extract total RNA from OVCA cells in vitro. Total RNA was extracted from tumor tissue using RNeasy Kit (Qiagen) and cDNA was prepared from 100 ng total RNA using Superscript III First Strand synthesis kit and oligo‐dT primers (Thermo Fisher Scientific). mRNA expressions were quantitated using CHOP (Hs01090850_m1), XBP1s (Hs03929085_g1), ATF3 (Hs00910173_m1), ATF4 (Hs00909569_g1), and DR5 (Hs00366278_m1) transcripts from Taqman probes (Life Technologies) in a LightCycler real‐time PCR machine (Roche Diagnostics, Indianapolis, IN). qPCR results were presented as means from three independent experiments and normalized to GAPDH (Hs02786624_g1) transcript. The comparative C_t_ formula was used in fold change calculations.

### 2.6 Annexin‐V apoptosis assay

OVCA cells were treated with ONC201 (20 µM) for different time points. Apoptosis Annexin‐V fluorescein isothiocyanate (FITC)/propidium iodide (PI) Kit (Beckman Coulter) were used for this analysis.^24^ The cells were washed by ice‐cold DPBS before harvest and resuspended the cell pellet in binding buffer following the addition of Annexin‐V and PI solution. Whole‐sample processing was performed keeping the sample tubes on ice. Samples were run three times to determine baseline and drug‐induced apoptosis. Apoptotic events were recorded as a combination of early apoptotic (Annexin‐V+/PI‐) and late apoptotic/dead (Annexin‐V+/PI+) events and data were presented as percent of Annexin‐V+ cells. In addition, the percentage of viable cells relative to vehicle controls was also recorded.

### 2.7 Caspase‐Glo 3/7 assay

To detect Caspase 3/7 activity Caspase‐Glo 3/7 assay kit (Promega Corporation) was utilized. Briefly, SKOV3 and VOA4627 cells were seeded in 96‐well white luminometer assay plates at a density of 4000 cells per well and incubated at 37°C for overnight. Cells were treated with ONC201 for 60 h. An equal volume of Caspase‐Glo 3/7 reagent was added into each well media following 1 h incubation on a shaker at room temperature in dark. The luminescence intensity was measured via a microplate reader (Synergy H1; BioTek) and calculated the intensity difference between vehicle control and treated ones as fold changes to control.

### 2.8 JC‐1 assay

JC‐1 assay was performed to assess Mitochondrial membrane potential (MMP). 400 µl of JC‐1 (1×) working dilution was added to 100 µl cell suspension and incubated for 15 min at a cell culture incubator. Samples were washed once in PBS, resuspended in complete medium, and analyzed on a Coulter XL Flow Cytometer equipped with an Argon laser (Beckman Coulter) JC‐1 fluorescence was analyzed examining dual parameter FL1(JC‐1 Green) versus FL2 (JC‐1 Red) histograms controlling for intact cells (based on size criteria). Cells exhibiting loss of mitochondrial potential display progressively increased JC‐1 green fluorescence with eventual loss of JC‐1 red fluorescence. Increases in JC‐1 Red/JC‐1 Green co‐positive and JC‐1 Green positive alone events relative to control samples represent increased loss of mitochondrial potential.

### 2.9 Western blot

The cells were seeded into 10 cm culture dishes and treated with desired drug dose for desired time points following harvest on the same time. At harvest, cells were washed with DPBS before lysis in a RIPA (Radio‐immunoprecipitation assay buffer) buffer along with protease and phosphatase inhibitors. Depending on the molecular weight of desired protein, 50–100 µg of protein was separated using respective percentage (8, 10, or 12%) of sodium dodecyl sulfate (SDS)—polyacrylamide gel following transferred onto polyvinylidene fluoride (PVDF) membranes. To confirm the successful protein transfer to PVDV membrane, Ponceau S staining solution (Sigma Aldrich) was used. The blots were then immunostained with the appropriate primary antibodies (1:500–1000) followed by appropriate Alexafluor secondary antibodies (1:5000). The immunoreactive proteins were visualized using the Odyssey Infrared Imaging System (Li‐Cor), as described by the manufacturer. Densitometric analysis was performed using NIH ImageJ software version 1.50i (https://imagej.nih.gov/ij/docs/menus/analyze.html). All western blot experiments were repeated at least in triplicate. Representative data are shown.

### 2.10 Human ovarian cancer xenograft model

Xenograft experiments were performed under the protocol (#17‐08‐0315) approved by the Ethics Committee for Wayne State University Institutional Animal Care & Use (WSU IACUC) with a project identification code (AMTEC Exp Ref # 3889). Eight weeks old female SCID mice, purchased from Charles River Laboratories (Wilmington, MA) were used to maintain serial passage of SKOV3 ovarian tumors in vivo. Initially, two mice were inoculated subcutaneously (SC) with 1 × 10^6^ cells bilaterally under the right and left axilla and were passed when the total tumor burden reached 1500 mg. To test the efficacy of ONC201 against SKOV3 tumors, 30 mg tumor fragments were implanted bilaterally SC in the axilla using a 12‐gauge trocar. Twelve mice were randomized into two groups (6 mice/group), a vehicle control and ONC201‐treated groups. ONC201 was dissolved in phosphate‐buffered saline (PBS) (diluent) and it was administered orally (PO) three days post implant at 125 mg/kg (0.2 ml/19 g mouse) once/week. Vehicle control mice received PBS (PO) on a matching volume and schedule. Tumor growth was assessed twice a week using a caliper and tumor volume (mm^3^ = mg) was determined using the formula *L* × *W* ^2^/2, where *L* is the length of the tumor and *W* is the width. Mice were euthanized after 72 h of last treatment at day 24 and tumors were harvested for molecular analysis. This study was carried out under the approval of the Wayne State University Animal Care and Use Committee (WSU IACUC) which strictly follows the recommendations of “The Guide for the Care and Use of Laboratory Animals” (National Science Foundation, 2011). WSU’s Animal Program is fully accredited by the American Association for Accreditation of Laboratory Animal Care (AAALAC). For the duration of the study, mice were housed at WSU within an environmentally controlled animal vivarium under 12 h light/dark cycles with veterinary care provided by the Division of Laboratory Animal Resources (DLAR).

### 2.11 Combinational studies

The cell viability was determined using CyQuant Cell. Briefly, 4000 cells per well in RPMI‐1640 medium were seeded into a flat‐bottomed 96‐well culture plate and allowed to attach overnight. For single drug treatment, the solutions were added for 72 h at a range of at least three to five concentrations to triplicate wells. For combination studies, IC_50_ dose of ONC201 were co‐treated with IC_50_ dose of Taxol, respectively, for 72 h. The combined effect was studied using the median effect analysis; the combination index (CI) was calculated using pooled data from three to five individual experiments each comprising at least three data points. The CI was calculated using Calcusyn software (V2).

### 2.12 Statistics

All the statistical analyses were performed using Microsoft Excel 2016. Data were expressed as the mean ± SD where needed. The significant differences between the vehicle and treated groups are determined using unpaired Student's *t* test. A *p*‐value ≤0.05 is presented as * and *p* ≤ 0.01 as **.

## 3 RESULTS

### 3.1 ONC201 inhibits cell growth and migration potential in HG and LG OVCA cell lines

**FIGURE 1:**
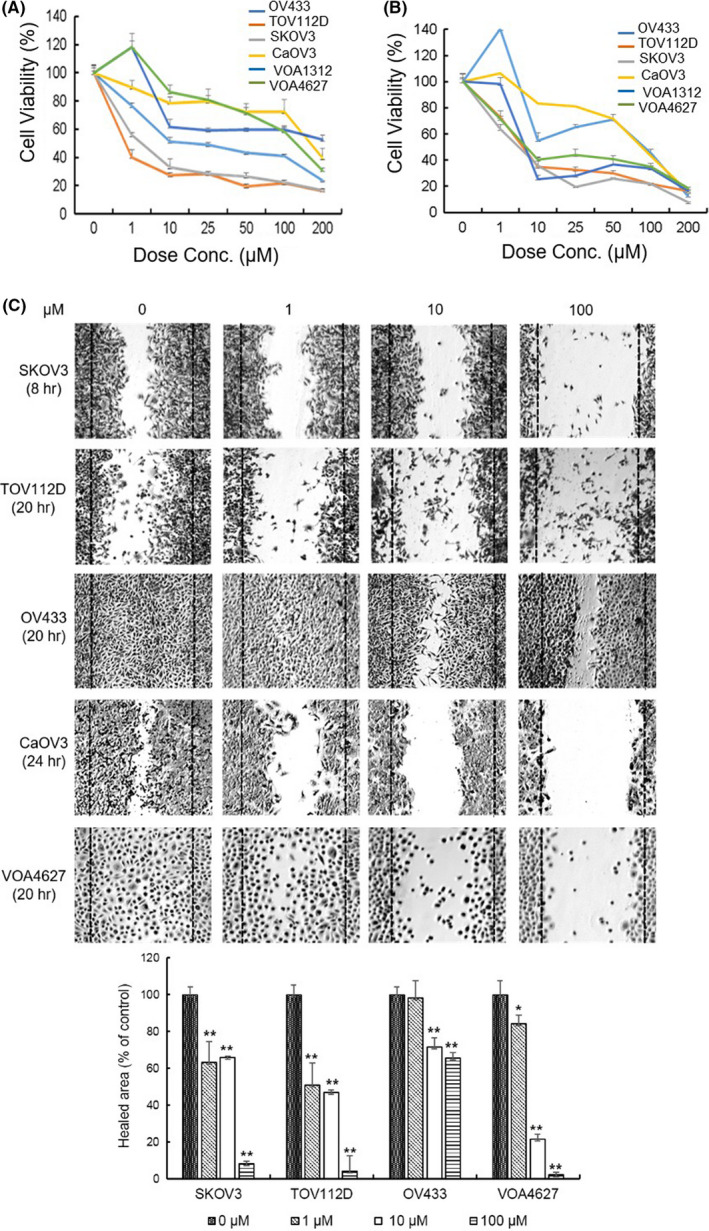
ONC201 inhibits viability of OVCA cells. (A and B) ONC201 reduces cell viability of SKOV3, OV433, CaOV3, TOV112D, VOA4627, and VOA1312 cells in a dose and time‐dependent manner. Cells were treated with either vehicle or drug at the indicated concentration (0/1/10/25/50/100/200 µM) for (A) 48 h and (B) 72 h MTT assay results are shown. The absorbance was read at 540 nm using an automated microplate reader. The percentage of cell viability was calculated to compare the vehicle group. Data were expressed as the percent cell proliferation relative to the control as mean ± SD from triplicate wells. (C) ONC201 inhibits OVCA migration. Cells were treated by ONC201 (0/1/10/100 µM) and migration of cells was evaluated via wound healing assay. Cells were seeded and left overnight. The following day a “wound” was created using 200 µl pipet tips and treated with ONC201. After incubation with ONC201 for 8–24 h depending on the cell line, cells were washed by DPBS and were stained with crystal violet. Dose‐dependent decreased cell migration was noted in all cell lines. A representative image from three independent experiments is shown (image magnification ×200). A *p*‐value ≤0.05 is presented as * and *p* ≤ 0.01 as **

To examine the activity of ONC201 in OVCA, HG (SKOV3, OV433, TOV112D, and CaOV3) and LG (VOA4627, VOA1312) OVCA cells were used. The MTT assay was used to identify the dose‐dependent effect of ONC201 at 48 and 72 h (Figure 1A and B, respectively). In particular, 10–25 μM of ONC201 inhibited the cell growth by at least ~50% in SKOV3, OV433, TOV112D, CaOV3, and VOA1312 cells at 72 h. Of note, not all cell lines were equally sensitive to ONC201 as demonstrated in Figure 1 although all demonstrated significant response. IC_50_’s further decreased at 96 h (data not shown). For direct evaluation of cell proliferation, trypan blue exclusion assay was also performed. Similar cell survival data were noted as in the MTT assays. Figure S1 depicts a representative figure for SKOV3 cells. ONC201 also inhibited migration of OVCA cells as demonstrated in wound‐healing migration studies in multiple cell lines (Figure 1C).

### 3.2 ONC201 downregulates PI3K/AKT and ERK/MEK signaling pathway in OVCA cells

**FIGURE 2:**
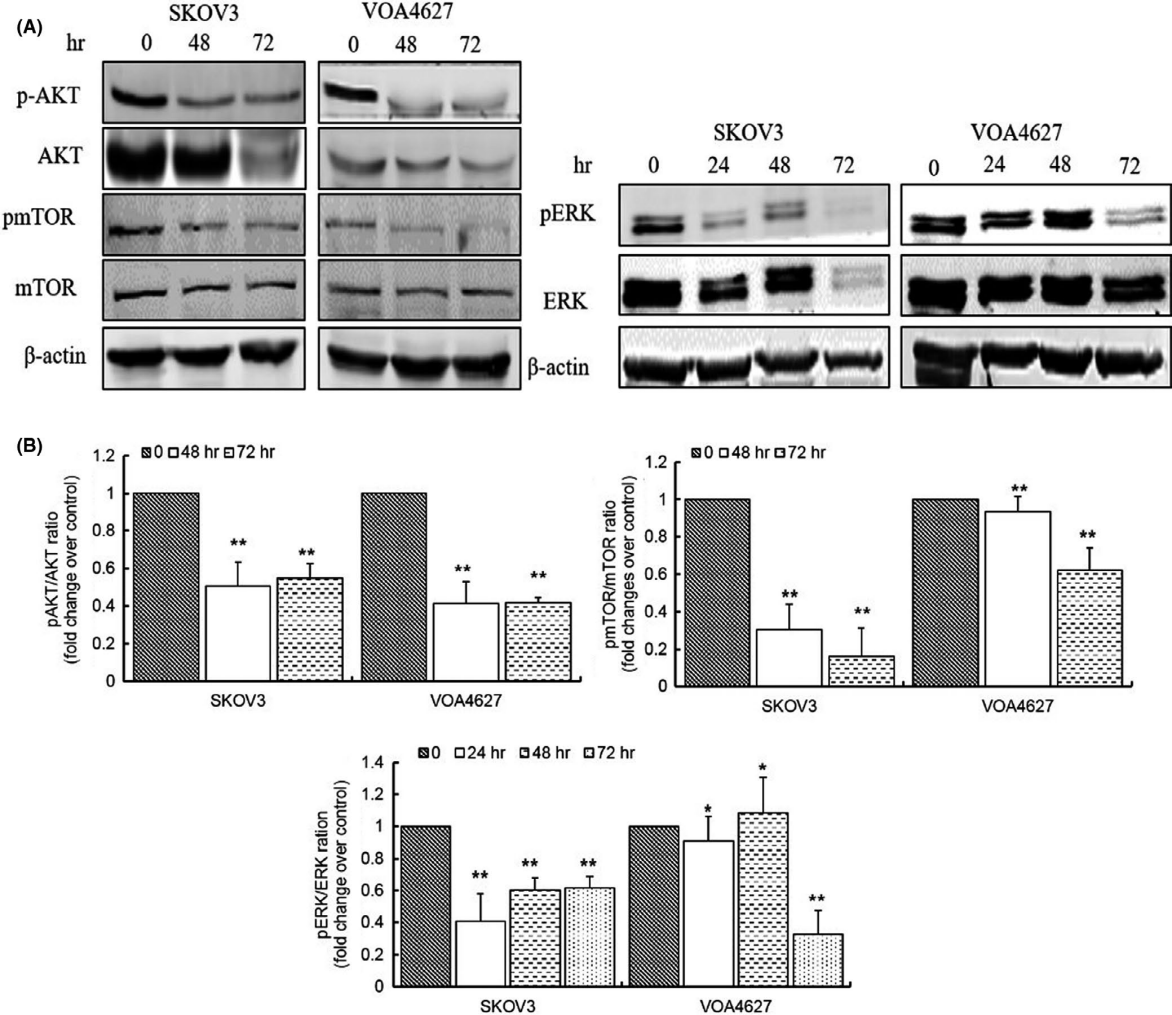
ONC201 downregulates PI3K/AKT and ERK/MEK signaling pathway. ONC201 (20 µM) inhibits AKT and ERK in high‐ and low‐grade OVCA cells. Lysates were collected from SKOV3 and VOA4627 cell lines at 48, 72, and 96 h and western blot was completed with indicated antibodies. A *p*‐value ≤0.05 is presented as * and *p* ≤ 0.01 as **

To elucidate the mechanism of ONC‐201, we initially evaluated two major signaling pathways (PI3K/AKT/mTOR, ERK/MEK) that had previously been described to be downstream of ONC201 inhibition.^25^ We observed treatment of OVCA cells with ONC201 downregulates both the PI3K/AKT and ERK/MEK signaling pathways (Figure 2). Notably, phosphorylated AKT decreased after 48 h treatment and total AKT expression was also decreased. Moreover, downstream of AKT, phosphorylated‐mTOR also decreased in response to ONC201. Phosphorylated ERK and MEK were also noted to be decreased in the ONC201‐treated cells.

### 3.3 ONC201 induces ER stress in OVCA cells

**FIGURE 3:**
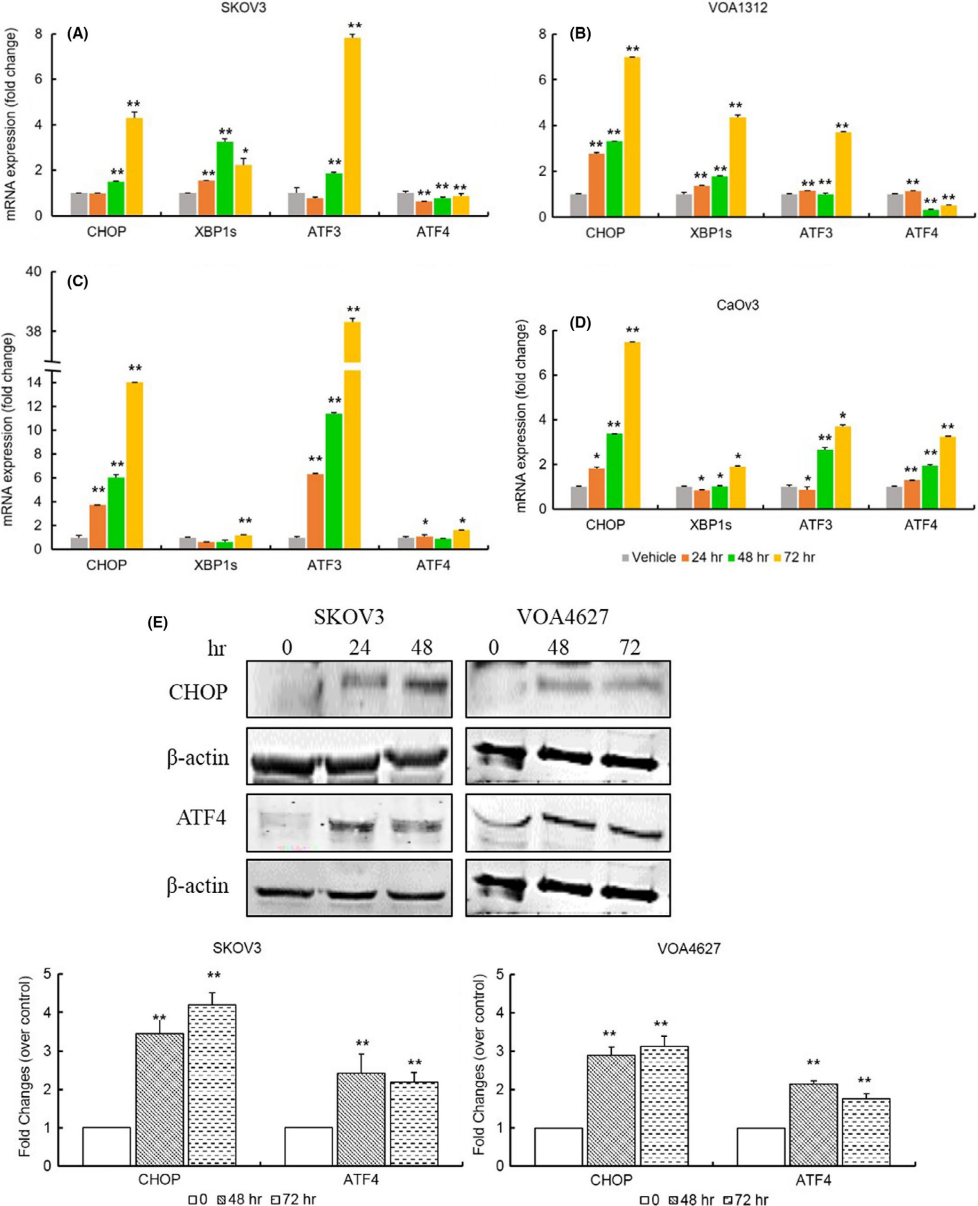
ONC201 activates ER stress. The expression of UPR‐related genes was evaluated by qPCR in SKOV3 (A), VOA1312 (B), OV433 (C), and CaOV3 (D). CHOP expression was induced 5–15 fold in all cell lines, suggesting that ONC201 prompts ER stress in human OVCA cells. (E) ONC201 (20 µM) upregulated protein expression of CHOP and ATF4. A *p*‐value ≤0.05 is presented as * and *p* ≤ 0.01 as **

To determine the activation of UPR in OVCA cells, we studied the expression of UPR‐associated genes via qPCR after treatment with ONC201. As noted above, both the IRE‐1 and PERK arms of the UPR are important in the response to stress. XBP‐s (spliced, active form of XBP‐1), downstream of IRE‐1 is important in the initial adaptive response to stress as is CHOP. CHOP and ATF3, both downstream of PERK/ATF4, are key to cell death induction via the UPR after prolonged activation. We noted CHOP expression increased ~5–15 fold in OVCA cell lines studied by 48–72 h depending on the cell line (Figure 3). By qPCR analysis, the level of ATF3 was also induced by ONC201 in all cell lines studied. To confirm the expression of CHOP and ATF4 at protein level, we performed western blotting and found that both CHOP and ATF4 were upregulated after 48 h of ONC201 treatment in both LG and HG OVCA. While ATF‐4 mRNA levels are not upregulated to a large extent, at their highest ~2 fold; the protein levels increase over time suggesting activation of the PERK arm of the UPR.

### 3.4 ONC201 induces apoptosis in HG and LG ovarian carcinoma cell lines

**FIGURE 4:**
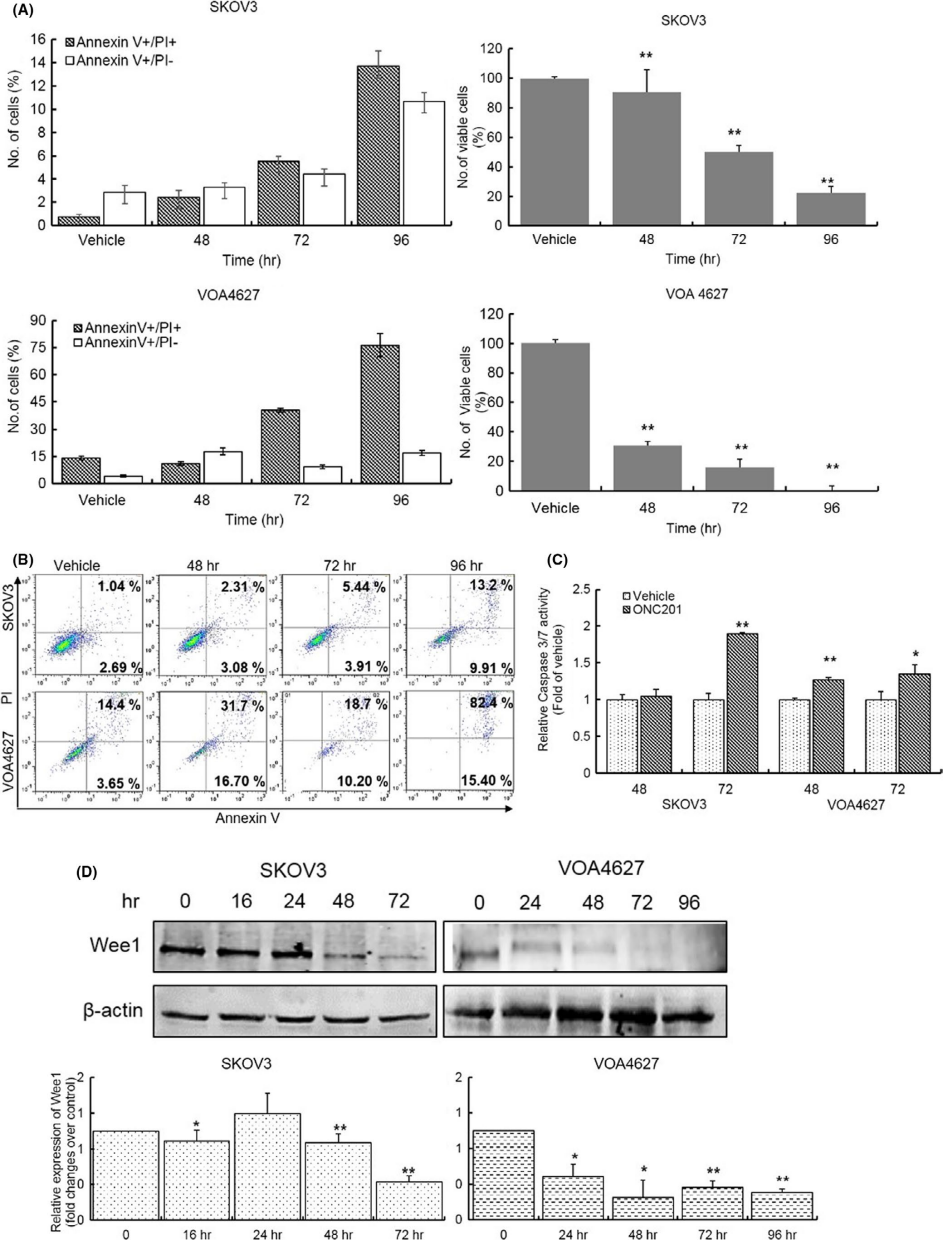
ONC201 promotes apoptosis both in high‐ and low‐grade OVCA cells. (A) SKOV3 and VOA4627 cells treated with or without ONC201 (20 µM) were double‐stained with Annexin V and PI, and then analyzed by flow cytometry. The percentages of viable cells (compared to vehicle) are shown. (B) Representative flow diagram of Annexin V‐PI staining. (C) Caspase3/7 activity was measured by Caspase‐Glo 3/7 assay. SKOV3 and VOA4627 cells were treated with ONC201 (20 µM) for 48, 72 h. Caspase3/7 activity was increased from 48 to 72 h. (D) ONC201‐treated lysates were collected from SKOV3 and VOA4627 cell lines and blotted with Wee1 antibodies. A *p*‐value ≤0.05 is presented as * and *p* ≤ 0.01 as **

To identify the effect of ONC201 on apoptosis, treated OVCA cells were double‐stained with Annexin V and PI, and the cellular apoptosis rates were determined by flow cytometry (Figure 4A and B). Treating cells with 20 µM of ONC201 increased both early apoptosis (Annexin V+/PI−) and late apoptosis (Annexin V+/PI+) subpopulations. Compared with vehicle‐treated cells, the total apoptosis rates of ONC201‐treated SKOV3 and VOA4627 cells increased in a time‐dependent manner. Respectively, there were 1.42, 2.5, and 6.22 fold increases in total Annexin V+ SKOV3 cells at 48, 72, 96 h. This observation was also confirmed by increased Caspase 3/7 cleavage/activation by ONC201 treatment (Figure 4C). Wee1 is rapidly cleaved by caspases to control entry into and exit out of mitosis.^26^, ^27^ In recent studies, Wee1 inhibition has been shown as a promising therapeutic target in lung cancer,^28^ hepatic cancer,^29^ and multiple myeloma.^30^ Considering this connection between UPR activation, Wee1 inhibition, and apoptosis, we examined Wee1 expression in OVCA cells. Interestingly ONC201 treatment also leads to downregulation of Wee1 expression, which may further promote apoptosis by leading to premature mitosis (Figure 4D).

### 3.5 ONC201 activates the intrinsic apoptotic pathway

**FIGURE 5:**
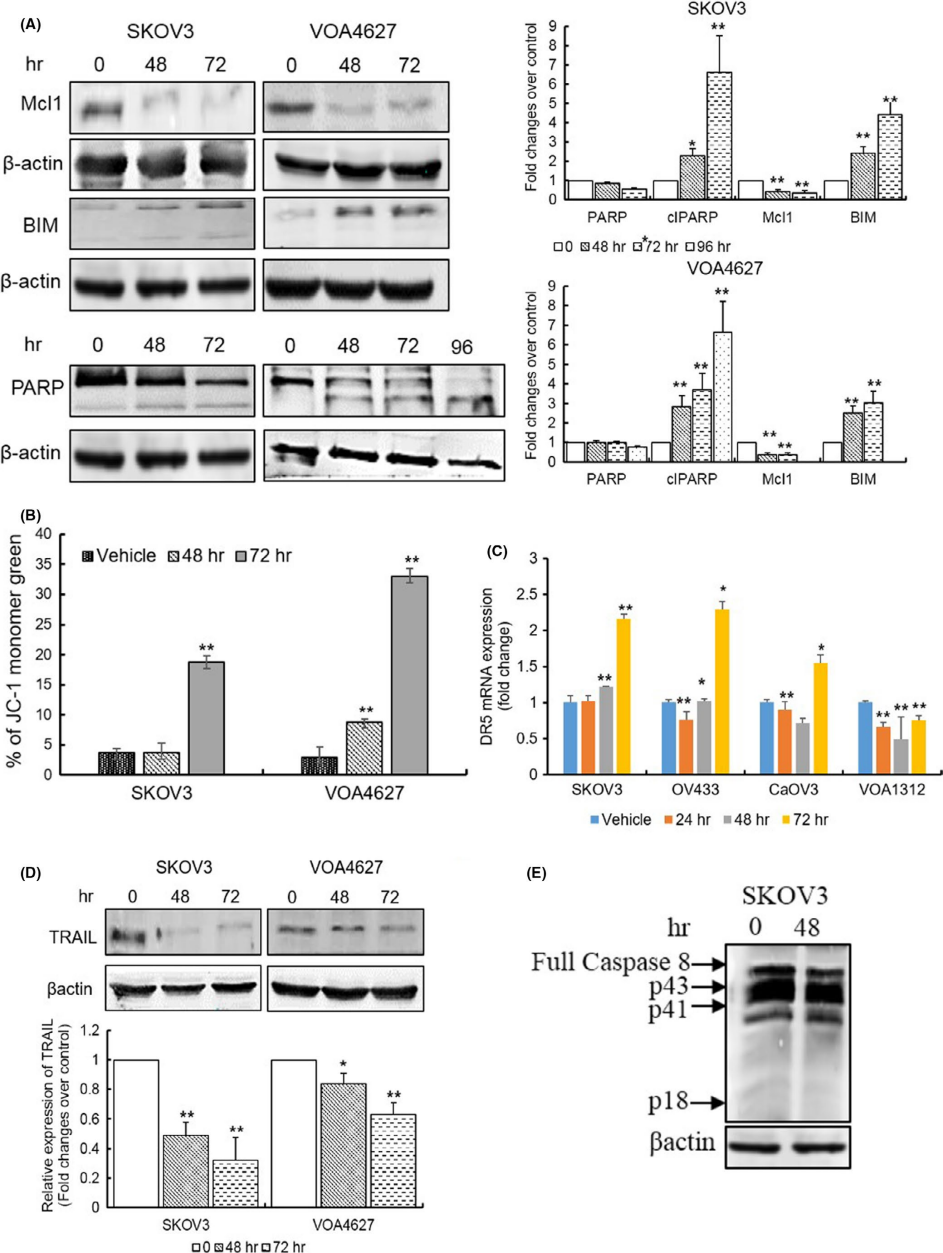
ONC201 leads cells to intrinsic rather than TRAIL‐induced cell death. (A) ONC201 leads to a loss of mitochondrial membrane potential (MMP). MMP was measured by JC‐1 assay. JC‐1 monomers (green) were increased by ONC201 (20 µM) treatment over time both in SKOV3 and VOA4627 cells. (B) After treating with ONC201 (20 µM) for 48 and 72 h, cells are harvested and cell extracts were analyzed by western blotting to detect the expression of cleaved PARP, BIM, and Mcl‐1. (C and D) ONC201 (20 µM) led OVCA cells to death via TRAIL‐independent pathway. Lysates were collected from SKOV3 and VOA4627 cell lines and blotted with TRAIL antibodies. Whereas, the expression of DR5 gene was confirmed by qPCR in SKOV3, OV433, CaOV3, and VOA1312. (E) ONC201 does not activate Caspase‐8. Caspase‐8 (p18/p10) was found intact both in ONC201 treated and control SKOV3 cells. A *p*‐value ≤0.05 is presented as * and *p* ≤ 0.01 as **

Furthermore, to identify whether ONC201 triggers apoptosis via the extrinsic (death receptor) or intrinsic (mitochondrial) pathway in human OVCA cells, both SKOV3 and VOA4627 cell lines were treated with ONC201 (20 μM) for 48 and 72 h. To determine if ONC201 leads to loss of mitochondrial membrane potential (MMP), a hallmark of intrinsic pathway‐mediated apoptosis, we performed JC‐1 probe staining via flow‐cytometry. ONC201 treatment induced a five and eightfold increase in the JC‐1 monomer shift, respectively, in SKOV3 and VOA4627 cells compared to vehicle‐treated cells confirming loss of MMP (Figure 5A). Moreover, we examined the expression of BIM (pro‐apoptotic protein), Mcl‐1 (anti‐apoptotic protein), and PARP cleavage. ONC201 treatment increased BIM, led to PARP cleavage and downregulated Mcl‐1 expression (Figure 5B). Finally, we studied the expression of TRAIL via western blotting and DR5 mRNA expression through qPCR as evidence of extrinsic pathway with some changes noted in DR5 at later time‐points (felt to be secondary to UPR) and a decrease noted in TRAIL protein (Figure 5C and D). ONC201 treatment, however, did not lead to cleavage of Caspase 8 at p18/p10 in SKOV3 and VOA4627 cells, indicating TRAIL‐independent apoptosis (Figure 5E and data not shown).

### 3.6 Weekly oral dose of ONC201 suppresses tumor growth in a xenograft mouse model

**FIGURE 6:**
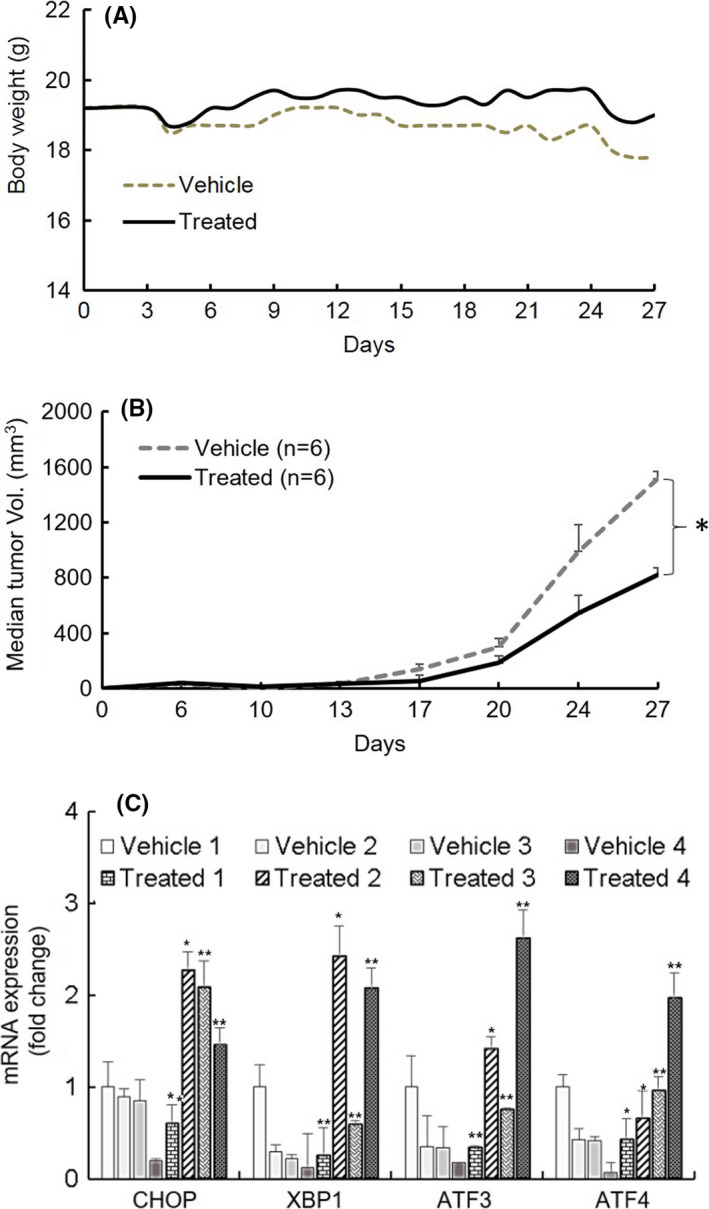
ONC201 treatment markedly reduces the growth of human ovarian cancer tumor xenografts. SCID mice (*n* = 6/group) with subcutaneous axillary SKOV‐3 xenograft tumors were treated with vehicle, ONC201 (125 mg/kg weekly) for 28 days and euthanized at 30 days. (A) Time course measurements of mouse weights, and (B) tumor volume at weekly intervals during treatments. (C) qPCR analysis of UPR‐related genes (CHOP, ATF3, ATF4, XBP1) expression in harvested tumors. Four separate tumor samples of each group were presented in the graph. Values are mean ± SEM; *p*‐value is 0.05 compare to vehicle group. Asterisk indicates significant differences at *p* = 0.05 by Student's *t* test

Lidia et al. characterized ovarian cancer cell lines as in vivo models for preclinical studies and utilized SKOV3 as representative for HG and CaOV3 and TOV112D as low tumorigenic models, based on their ability to form tumors in subcutaneous models.^31^ Considering tumorigenicity of SKOV3 and our in vitro data, we established an SKOV3 axillary xenograft model to evaluate ONC201’s in vivo efficacy. SCID mice with SKOV3 axillary xenografts were treated via oral gavage with either vehicle or ONC201 (125 mg/kg) on a Q7d × 4‐week schedule. Treatment with ONC201 led to tumor growth inhibition as a single agent compared to vehicle ones with stable body weight over the treatment period and no adverse events within the ONC201 treated versus control mice (Figure 6A). After 72 h of last treatment, we cut off the data at 28 days where the median tumor burden reached 1519 mm^3^ in the vehicle (control) group and 821 mm^3^ in the ONC201‐treated group, an approximate 50% reduction in tumor volume (Figure 6B). To evaluate whether ONC201 treatment affects UPR‐related gene expression in vivo, a portion of the control mice (after the 28 days trial was completed) were treated 24 h prior to sacrificing the mice with oral ONC201. Mice were sacrificed and RNA was extracted from control and ONC201‐treated tumors. qPCR was performed demonstrating an induction of CHOP in the tumors of the treated mice when compared to the control mice (Figure 6C).

### 3.7 ONC201 synergizes with conventional chemotherapy in a highly resistant OVCA cell line

In order to determine whether ONC201 sensitizes cells to conventional chemotherapy, we examined the combined action of ONC201 with Taxol in OV433, a highly platinum‐resistant cell line. The effect of ONC201 and Taxol treatment on cell viability were observed separately and in combination. Taxol was chosen given its use in platinum‐resistant patients and prior data in our lab demonstrating UPR induction with Taxol (data not shown). The respective IC_50_ values of ONC201 and Taxol were 32.3 µM and 0.065 µM in the OV433 cells. The activity of ONC201 in combination with Taxol was assessed using drug concentrations extrapolated from individual IC_50_ values. The combination index (CI) was used as a measure of synergy (CI < 1), addition (CI = 1), and antagonism (CI > 1). In OV433, we found synergy between ONC201 and Taxol with a CI =0.117 with a significant reduction in the respective IC_50_’s >30‐fold for both drugs (Table 1).

**TABLE 1:** Combination index (CI) analyses in OV433 cells

OVCA cell line	OV433	
Drug (s)	IC_50_ alone (µM)	IC_50_ in combo (µM)
ONC201	32.3	1.89
Taxol	0.0653	0.00379
CI		**0.117**

Cells were treated for 72 h with multiple doses of both ONC201 and Taxol. The IC_50_ alone and in combination was calculated in addition to the CI using calcusyn software. CI  = 1, additivity; CI > 1, antagonism; CI < 1, synergy.

Bold indicates significant synergy.

## 4 DISCUSSION

ONC201 is a first‐in‐class small molecule selective orally bioavailable dopamine D2‐like receptor (DRD2) antagonist that is in Phase I‐II clinical trials in select cancers and has been well tolerated with minimal toxicity noted in these human trials.^32^, ^33^, ^34^, ^35^ Given ovarian cancer has >60% DRD2 surface expression it was felt that ONC201 would be an excellent candidate agent to study in this setting.^19^, ^25^, ^33^ From earlier in vitro and in vivo investigations, it has been noted that ONC201 has minimal anti‐proliferative effects in normal fibroblast and multiple different types of epithelial cells that are non‐apoptotic in nature. The normal cells completely recover upon drug removal, unlike cancer cells, and therefore this drug was felt to have a high therapeutic index.^34^, ^36^, ^37^

Here, we report that ONC201 demonstrates anti‐cancer efficacy both in high‐grade (type II, HG‐OVCA) and the less common slow‐growing low‐grade (type I, LG‐OVCA) cancers through downregulation of pro‐survival pathways and activation of the UPR, both of which ultimately lead to cell death. Our data further indicate that ONC201 suppresses cell viability and migration potential in OVCA cells regardless of platinum sensitivity. The IC_50_ at 72 h are ranging from 10 to 20 µM in resistant (SKOV3) and intermediate (OV433, TOV112D, VOA4627, and VOA1312) cell lines in terms of sensitivity to platinum as defined by previous publications.^38^, ^39^ Although not all cell lines were equally sensitive at 48 or 72 h, at 96 h, for slower growing cells, the IC_50_ was even lower into a range achievable in vivo (data not shown). Given this, different sensitivity to ONC201 appears to be related to the doubling time of the specific cell lines in question. For example, CaOV3 cell lines have a doubling time of 68–72 h ^40^, ^41^ as compared to other HGOVCA cell lines studied. Similarly, LG‐OVCA cell lines with longer doubling time required longer exposure to the drug (i.e., 96 h).

In agreement with our in vitro findings, we observed that once‐weekly dosing of ONC201 at 125 mg/kg dose reduces the tumor burden by ~50% in a xenograft mice model with no evidence of weight loss or other toxicity in the mice studied.

HG‐OVCA rely on the PI3 K/AKT/mTOR pathway with at least 50% of all OVCA demonstrating reliance on this pathway. Alterations or activation of the PI3K/AKT pathway lead to its constitutive activation, thereby leading to cell survival, proliferation, immune evasion, and angiogenesis.^42^, ^43^, ^44^ In LG‐OVCA cells, modulation and inhibition of both the PI3K/mTOR and MEK/ERK pathways have also provided significant improvement in outcomes for patients, however current agents are quite toxic and none are yet FDA approved.^42^, ^45^, ^46^ We observed ONC201 leads to dephosphorylation of AKT, mTOR, and ERK in both HG and LG‐OVCA lines. It is likely that direct inhibition of these pathways leads to cellular stress which activates the UPR. In addition, activation of the UPR itself has been shown to further inhibit these pro‐survival pathways with prolonged activation.^47^ ONC201 has been shown previously to induce the UPR in cancer cell lines in vitro including breast and glioblastoma.^21^, ^36^, ^48^ In OVCA, this mechanism has not been elucidated. In the current experiments, we found that ONC201 treatment preferentially upregulates the PERK‐ATF4‐CHOP arm of the UPR in all cell lines studied. This is the pro‐apoptotic arm of the UPR. This activation is via phosphorylation of PERK at serine 51, which results in attenuation of protein translation and upregulation of the transcription factor ATF4.^49^ While no significant change in ATF4 levels at the transcriptional/mRNA levels were noted, we did observe a significant upregulation at the protein level, suggesting either a decrease in protein turnover or an increase in translation. Prior studies with other tyrosine kinase inhibitors, such as Sorafenib, have shown similar post‐transcriptional regulation of ATF4^50^ and it is likely that ONC201 has a similar mechanism, however this will need to be studied in future experiments.

Recent publications have suggested that ONC201 may specifically activate the intrinsic pathway of apoptosis in certain cell types rather than dual activation of both the intrinsic and extrinsic apoptotic arms or purely TRAIL‐mediated apoptosis.^19^, ^20^, ^51^, ^52^ CHOP is downstream of ATF4 and regulates several anti‐apoptotic (BCL2, BCL‐XL, MCL‐1, and BCL‐W) and pro‐apoptotic (BAK, BAX, BOK, BID, BIM, BAD, BIK, NOXA, and PUMA) BCL‐2 family members via downregulation and upregulation, respectively, in sustained UPR induction. Ultimately, these changes lead to intrinsic pathway activation via mitochondrial depolarization. In this study, we documented direct depolarization and loss of MMP with ONC201 treatment and cleavage of Caspase 3/7 and PARP along with regulation of multiple pro‐apoptotic factors. While we also observed that the upregulation of CHOP leads to the induction of death receptor (DR5) it downregulates the expression of the ligand for DR5, TRAIL. Additionally, caspase 8, the initiator caspase of the extrinsic pathway^20^, ^48^, ^53^ is not cleaved by ONC201. These findings suggest ONC201 induced apoptosis is via the intrinsic pathway alone in both HG and LG‐OVCA cells.

Given our prior findings that Taxol induces UPR in OVCA cell lines (data not shown), we hypothesized that the combination of this agent with ONC201 would further potentiate the activity of each drug. Additionally, single‐agent weekly Taxol has demonstrated both anti‐angiogenic and pro‐apoptotic characteristics^54^, ^55^, ^56^ and is highly active in recurrent, platinum‐resistant OVCA as a single agent.^57^ In fact, Taxol is now being studied as the backbone therapy in combination with multiple targeted agents because of these characteristics. We found that ONC201 and Taxol synergized in a highly resistant cell line, OV433 (CI = 0.117) with significant reductions in the IC_50_’s of both drugs. This combination specifically is therefore very promising, and these findings suggest that combining agents that induce the UPR in OVCA is a viable means to overcome platinum resistance.

## 5 CONCLUSIONS

**FIGURE 7:**
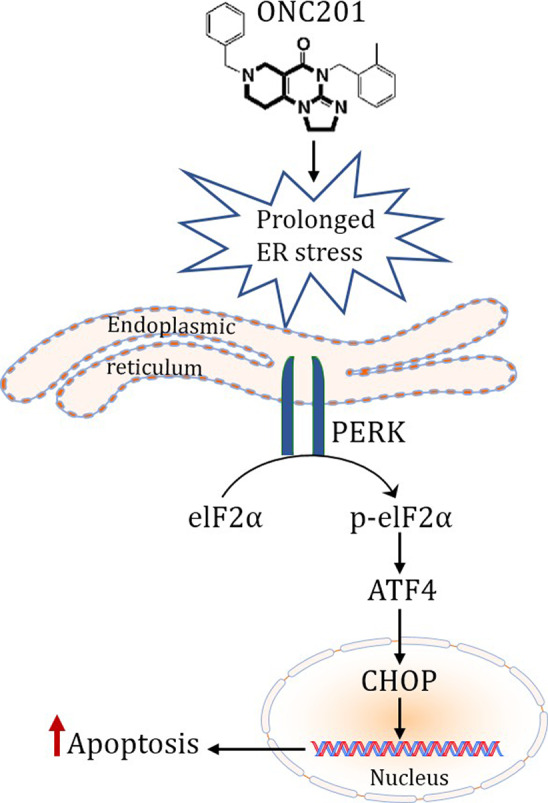
ONC201 increases endoplasmic reticulum stress in both high‐grade and low‐grade ovarian cancer cells; overwhelming pro‐survival signals and activating the pro‐death arm of the unfolded protein response pathway. In addition, a weekly oral dose of ONC201 lowers the tumor burden in mice. This is a promising therapeutic agent in OVCA treatment and should be considered for clinical translation

ONC201 is a well‐tolerated, oral agent currently in use in clinical trials with limited noted toxicity. Our current findings suggest that ONC201 could be a potent therapeutic candidate as a single agent and in combination with approved therapies for both high‐grade and low‐grade ovarian cancer patients (as summarized in the graphical abstract, Figure 7). Additionally, drugs that preferentially induce the pro‐apoptotic arm of the UPR should be investigated further as a means to overcome platinum resistance in ovarian cancer.

## Supporting information


Figure S1

## Data Availability

The data that support the findings of this study are available from the corresponding author upon reasonable request.
