# Supplementary figures and images for: ONC201 induces the unfolded protein response (UPR) in high‐ and low‐grade ovarian carcinoma cell lines and leads to cell death regardless of platinum sensitivity
Source: Cancer Med. 2021 May 1;10(10):3373–87. doi: 10.1002/cam4.3858 (PMC8124100; doi:10.1002/cam4.3858)

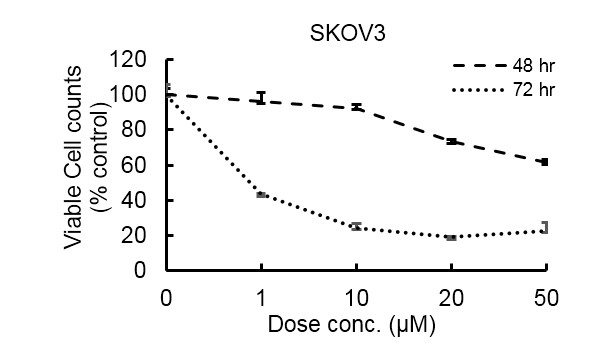

Supplement: Supplementary file 1 — Figure S1 [file CAM4-10-3373-s001.jpg]
